# Evaluation of Waste Anesthetic Gas in the Postanesthesia Care Unit within the Patient Breathing Zone

**DOI:** 10.1155/2015/354184

**Published:** 2015-11-26

**Authors:** Kenneth N. Hiller, Alfonso V. Altamirano, Chunyan Cai, Stephanie F. Tran, George W. Williams

**Affiliations:** ^1^Department of Anesthesiology, The University of Texas Medical School, Houston, TX 777030, USA; ^2^Division of Clinical and Translational Sciences, Department of Internal Medicine, The University of Texas Medical School, Houston, TX 777030, USA; ^3^Departments of Anesthesiology and Neurosurgery, The University of Texas Medical School, Houston, TX 777030, USA

## Abstract

Potential health hazards from waste anesthetic gases (WAGs) have been a concern since the introduction of inhalational anesthetics into clinical practice. The potential to exceed recommended exposure levels (RELs) in the postanesthesia care unit (PACU) exists. The aim of this pilot study was to assess sevoflurane WAG levels while accounting for factors that affect inhalational anesthetic elimination. In this pilot study, 20 adult day surgery patients were enrolled with anesthesia maintained with sevoflurane. Following extubation, exhaled WAG from the patient breathing zone was measured 8 inches from the patient's mouth in the PACU. Maximum sevoflurane WAG levels in the patient breathing zone exceeded National Institute for Occupational Safety and Health (NIOSH) RELs for every 5-minute time interval measured during PACU Phase I. Observed WAGs in our study were explained by inhalational anesthetic pharmacokinetics. Further analysis suggests that the rate of washout of sevoflurane was dependent on the duration of anesthetic exposure. This study demonstrated that clinically relevant inhalational anesthetic concentrations result in sevoflurane WAG levels that exceed current RELs. Evaluating peak and cumulative sevoflurane WAG levels in the breathing zone of PACU Phase I and Phase II providers is warranted to quantify the extent and duration of exposure.

## 1. Introduction

Potential health hazards from waste anesthetic gases (WAGs) have been a concern since the introduction of inhalational anesthetics into clinical practice. In 1977 the National Institute for Occupational Safety and Health (NIOSH) established a recommended relative exposure limit (REL) for halogenated anesthetic agents (without concomitant nitrous oxide) of 2 parts per million (ppm) for a period of time not to exceed 1 hour [1]. Standards vary in other countries. Great Britain, for example, has established an 8-hour time-weighted average exposure limit of 50 ppm for isoflurane. NIOSH recommends and The Joint Commission requires [2] active WAG scavenging systems in all anaesthetizing locations. In other locations, the Occupational Safety and Health Administration (OSHA), which is the relevant federal regulatory agency in the United States, recommends that WAG exposure be kept “to the lowest practical level” [3].

Reducing WAG exposure via active scavenging in all anaesthetizing locations is considered standard practice. Extensive research has failed to identify adverse health effects of trace anesthetic exposure commonly experienced in modern, scavenged operating rooms [4, 5]. However, recent studies observed an increased risk of spontaneous abortion and infertility in female veterinarians working in operating rooms without scavenging devices [6, 7]. The risks of preterm delivery (odds ratio 2.80) and spontaneous abortion (odds ratio 2.49) were significantly higher in women exposed to anesthetic gases working without scavenging devices compared to unexposed women or women protected by scavenging equipment [6, 7].

Although the PACU has not traditionally been considered a location that requires active scavenging, the potential to exceed recommended NIOSH WAG RELs exists. In 1998, Sessler and Badgwell found that WAG concentrations within the nurse breathing zone (described as a zone 8 inches directly in front of the mouth) exceeded RELs in 37% and 87% of patients given isoflurane and desflurane, respectively [8]. More than half of these patients received nitrous oxide in addition to inhalational agent and remained intubated on arrival to the PACU.

The aim of this pilot study is to assess sevoflurane WAG levels while taking into account factors that affect inhalational anesthetic elimination. This study measures WAG levels in the breathing zone of patients who (1) received sevoflurane without nitrous oxide, (2) were extubated in the operating room, and (3) recovered in a PACU that meets NIOSH engineering standards.

## 2. Methods

This observational pilot study was conducted in the PACU at Memorial Hermann Hospital-Texas Medical Centre, Houston, TX, USA. NIOSH-mandated engineering requirements for ventilation in the PACU are 6 air exchanges per hour, of which 2 must be fresh air [1] (air exchange requirements for the operating room are higher). Hospital engineers verified PACU ventilation to be a minimum of 10 air exchanges per hour, of which 4 are fresh air. Relative humidity was maintained within required parameters. After obtaining Institutional Review Board (IRB) approval and participant consent, 20 adult day surgery patients meeting study criteria which require them to remain in PACU for at least 1 hour were enrolled.

In the operating room, anesthesia was induced intravenously with propofol and maintained with a mixture of sevoflurane, oxygen, and air. Forced air warmers were used for all patients. Intraoperative temperature was measured by a nasopharyngeal temperature probe. Opiates were titrated to spontaneous respiratory rate toward the end of the anesthetic, the sevoflurane was turned off (rather than tapered), and patients were extubated at an end-tidal sevoflurane concentration of 0.2%. Intraoperative temperature and end-tidal sevoflurane concentration were recorded every 10 minutes from anesthetic induction through tracheal extubation in the operating room. Patients were transported to the PACU within <20 minutes with supplemental oxygen by simple face mask at a flow rate of 8 liters per minute.

Supplemental oxygen was continued at the same flow rate in the PACU. Exhaled WAG from the patient breathing zone was measured with a portable, calibrated Miran 1B infrared spectrophotometer (Thermal Fisher Scientific, Waltham, Massachusetts). Its accuracy is ±10% of the measured value with a usable range of 0.03–100 ppm. The response time for a single wavelength is 20 seconds. A wand attached to the Miran analyzer was positioned 8 inches directly in front of the mouths of patients when scanning during the first hour of the PACU Phase I recovery period. Patient age, gender, height, weight, body mass index (BMI), surgical procedure, procedure length, and intraoperative fluids given were documented. Blood pressure, heart rate, respiratory rate, and time of supplemental oxygen administration were recorded throughout Phase I recovery period.

### 2.1. Statistical Methods

Continuous variables with a normal distribution were reported as mean ± standard deviation (SD) and continuous variables with skewed distribution were summarized as median and interquartile range. For longitudinal maximum WAG value, we applied a generalized estimating equation method to evaluate its change over time. We included the covariates of time and its quadratic term in the regression model. All statistical analyses were performed using SAS 9.3 (SAS Institute Inc., Cary, NC), and a *p* value < 0.05 was considered significant.

## 3. Results

**Figure 1:**
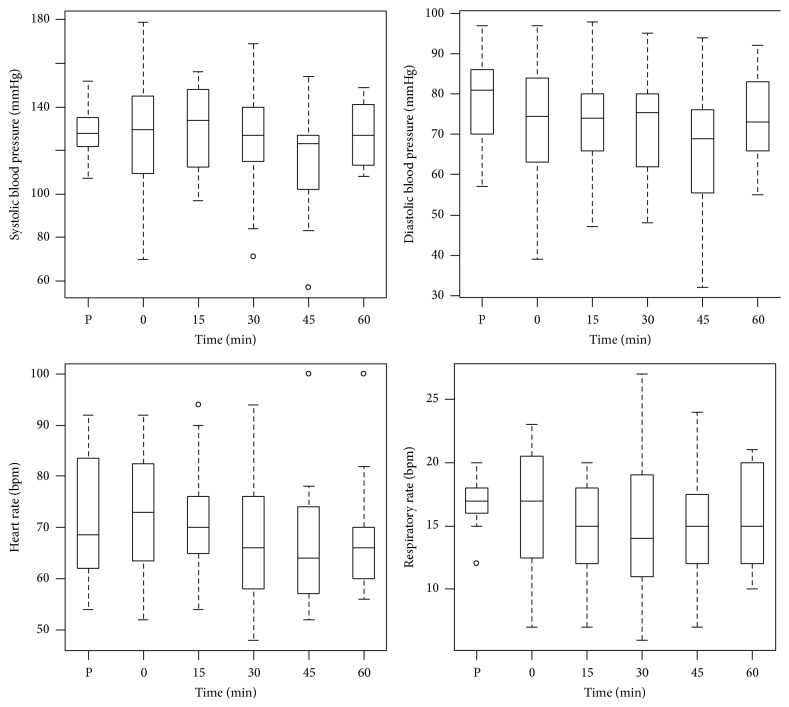
Preoperative vital signs (P) plotted beside boxplots of vital signs collected at 15 min time intervals in the PACU. There were no significant differences between preoperative and postoperative values.

Table 1 illustrates the age, height, weight, BMI, and temperature data collected from included patients. The median anesthetic duration and minimum alveolar concentration (MAC) hours were 100 minutes and 2.1, respectively. A MAC hour reflects the calculation of total volatile anesthetic consumption between patients and is calculated by dividing the mL of volatile anesthetic consumed by the duration of 1 MAC anesthesia, as described by Sessler and Badgwell and Tyagi et al. [8, 9].  Figure 1 shows minimal differences between preoperative vital signs (e.g., systolic blood pressure, diastolic blood pressure, heart rate, and respiratory rate) and their corresponding median PACU Phase I values.

**Figure 2:**
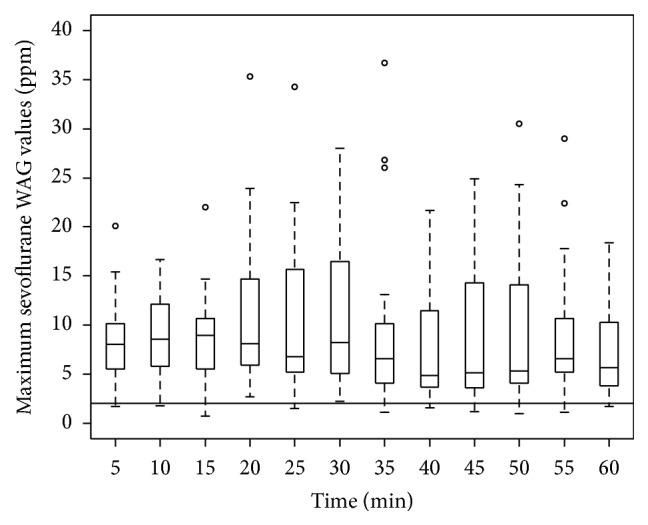
Box and whisker plot of maximum sevoflurane WAG values at 5 min intervals. The horizontal line at 2 ppm indicates the recommended NIOSH RELs.

Figure 2 shows that maximum sevoflurane WAG levels in the patient breathing zone exceeded RELs for every 5-minute time interval measurement during PACU Phase I recovery in all patients. WAG values in the first 23 minutes reflect simultaneous administration of supplemental oxygen by simple face mask. During this time period, our data likely underestimated the actual exhaled WAG values since they were diluted by 8 liters per minute oxygen flow. As reported in Figure 2, no statistically significant changes were detectable in the maximum WAG values over time.

## 4. Discussion

WAG levels in the patient breathing zone exceed RELs for healthcare workers throughout the entire measured time period. These nonexponential WAG changes can be explained by context-sensitive decrement times for inhalational anesthetics [10]. The majority of sevoflurane elimination occurs through the lungs since only 2–5% undergoes metabolism [11]. Eger and Shafer (2005) demonstrated that anesthetic solubility in blood and tissue, duration of anesthesia, and cardiac output can predict decrements from vessel rich group (VRG) anesthetic concentrations [10]. The authors showed that less soluble agents have the quickest washout, though the effect is less pronounced as anesthetic duration increases [10]. Behne et al. (1999) demonstrated in a pharmacokinetic study that the longer the duration of anesthesia, the greater its accumulation in muscle and fat [11]. This is reflected clinically as increased time requirement to achieve a desired decrement in VRG concentration [10, 12]. Eger and Shafer (2005) also illustrated the effect of cardiac output on sevoflurane washout; increasing cardiac output decreased the time for sevoflurane washout from the VRG [10]. If ventilation, cardiac output, and tissue perfusion remain constant (which can be inferred in our study since preoperative and postoperative vital signs are comparable, Figure 1), then the rate of washout and clinical recovery (MAC-Awake) [13] would be determined by the blood solubility of the volatile agent and the duration of exposure, that is, the “context” in context-sensitive decrement [10]. The rate of washout of sevoflurane for a given patient in our study became dependent solely on the duration of anesthetic exposure.

WAGs are expressed in ppm. Anesthetic gas is delivered as percent concentration; for example, 100% concentration of inhalational agent is 1,000,000 ppm. Patients in our study were extubated at an end-tidal sevoflurane concentration of 0.2%, which is 2000 ppm. Assuming a constant cardiac output of 6 liters per minute, 92% of sevoflurane would be eliminated from the VRG after 25 minutes [10]. Elimination of 92% of 2000 ppm would leave 184 ppm in the VRG. Elimination of 95% of sevoflurane under equivalent conditions would take 75 minutes and leave 100 ppm. The current WAG REL of 2 ppm corresponds to an end-tidal concentration of 0.002%, a criterion that requires a decrement in inhaled anesthetic of 99.998%.

Sessler and Badgwell (1998) attributed elevated WAG levels in the nurse breathing zone to insufficient fresh air ventilation [8]. Our current knowledge of inhaled anesthetic pharmacokinetics provides an alternate explanation for their nonexponential WAG decrease. These pharmacokinetic principles also predict that WAG levels in the patient breathing zone will exceed RELs extending into the PACU Phase II time period in our study, which is what we observed. Maximum WAG levels were utilized for the purposes of measurement and discussion as NIOSH guidelines specifically limit maximum WAG exposure while not addressing minimum or average values [1].

There were some important limitations in our pilot study. First, we did not measure WAG levels in the nurse breathing zone. In contrast to Sessler and Badgwell [8], our study controlled for variables that affect elimination of volatile agent: (1) administering sevoflurane without nitrous oxide to avoid the second gas effect during emergence [14], (2) observing respiratory and cardiovascular vital signs as indirect measures of ventilation and cardiac output, (3) accounting for supplemental oxygen delivery, (4) extubating all patients in the operating room, and (5) ensuring that recirculating ventilation in the PACU meets NIOSH standards. Even with these limitations, WAG of desflurane exceeded nurse breathing zone RELs in 87% of patients in their study [8]. In addition to the modifications described above, the duration of anesthesia (100 versus 60 min) and MAC hours (2.1 versus 1.5) was significantly higher in our study than in Sessler and Badgwell [8]. Taking into account all of these factors, inhalational agent pharmacokinetics predict that an even greater percentage of sevoflurane WAG measurements in the nurse breathing zone would exceed RELs in our study compared to theirs [10]. A second limitation of our study is that sevoflurane was not tapered toward the end of the anesthetic. Tapering would decrease the time required to achieve a desired decrement in VRG concentration and result in lower measured WAG levels [10]. Finally, WAG levels can be expected to fall off with greater distance from the patient secondary to factors such as PACU air flow, PACU bed layout, and proximity of patients one to another. Further study measuring WAG level in the patient breathing zone while simultaneously measuring WAG level in the caregiver breathing zone could serve to clarify the expected relationship between the two said levels if PACU air turnout is set to meet US standards.

## 5. Conclusions

Our study fills a knowledge gap in the existing literature by documenting elevated sevoflurane WAG levels in the patient breathing zone in the PACU.

Developing cogent risk mitigation strategies will require additional study. Evaluating peak and cumulative WAG levels in the patient breathing zone while simultaneously measuring WAG levels in the breathing zone of healthcare providers is warranted to quantify exposure risk relative to the duration of anesthesia.In the meantime, it may be prudent to minimize healthcare worker exposure to the patient breathing zone when practical.

## Figures and Tables

**Table 1:** Morphometric characteristics (*n* = 20).

Age (years)	48 ± 15
Height (cm)	173 ± 10
Weight (kg)	83 ± 13
BMI (kg/m^2^)	28 ± 4
Temporal artery temperature (°C) on PACU arrival	36 ± 1
Anesthetic duration (min), median (Q1, Q3)	100 (75, 165)
Minimum alveolar concentration (MAC) hours	2.1 ± 0.4
Supplemental O_2_ administration time in PACU (min)	23 ± 18
Phase I PACU time (hours)	1.6 ± 0.6

Unless otherwise noted, data are presented as means ± SD. Q1, 1st quartile; Q3, 3rd quartile.

## Abbreviations

WAGs: Waste anesthetic gases
PACU: Postanesthesia care unit
RELs: Recommended exposure levels
NIOSH: National Institute for Occupational Safety and Health
OSHA: Occupational Safety and Health Administration
IRB: Institutional Review Board
SD: Standard deviation
VRG: Vessel rich group
MAC: Minimum alveolar concentration
BMI: Body mass index.
